# Supplementary material for: The proximal N-terminus of IRAG is required for potentiation of HCN4 channels
Source: bioRxiv. 2026 Apr 23:2026.04.20.719713. Preprint. [Version 1] doi: 10.64898/2026.04.20.719713 (PMC13131473; doi:10.64898/2026.04.20.719713)
Supplement: Supplement 1 [file NIHPP2026.04.20.719713v1-supplement-1.pdf]

bioRxiv preprint doi: https://doi.org/10.64898/2026.04.20.719713; this version posted April 23, 2026. The copyright holder for this preprint (which was not certified by peer review) is the author/funder, who has granted bioRxiv a license to display the preprint in perpetuity. It is made available under aCC-BY 4.0 International license.

SUPPLEMENTARY TABLES

Supplementary Table 1

2-Way ANOVA			
condition	Control	IRAG	IRAG vs. Control
HCN4	-117.1 ± 1.4 (15)	-104.3 ± 2.3 (11)	p < 0.001
+ cAMP	-104.8 ± 1.4 (8)	-105.0 ± 2.8 (10)	p = 0.954
	p < 0.001	p = 0.829	

Supplementary Table 1 Legend:
Two-way ANOVA with Bonferroni post hoc test for multiple comparisons. Mean values are reported ± SEM with n listed in parentheses. P values below columns represent the comparison between the two above values; P values at the end of rows represent the comparison between the two values to the left.

bioRxiv preprint doi: https://doi.org/10.64898/2026.04.20.719713; this version posted April 23, 2026. The copyright holder for this preprint (which was not certified by peer review) is the author/funder, who has granted bioRxiv a license to display the preprint in perpetuity. It is made available under aCC-BY 4.0 International license.

Supplementary Table 2

2-Way ANOVA							
condition	control	IRAG Δ1-43	vs. control	IRAG 1-43-cit	vs. control	10uM IRAG 1-43	vs. control
HCN4 WT	-117.1 ± 1.4 (15)	-116.1 ± 0.9 (9)	p = 1.000	-109.2 ± 2.3 (16)	p = 0.008	-103.9 ± 4.4 (7)	p < 0.001
+ cAMP	-104.8 ± 1.4 (8)	-101.9 ± 1.5 (9)	p = 1.000	-105.1 ± 1.2 (11)	p = 1.000	-105.8 ± 2.7 (7)	p = 1.000
	p < 0.001	p < 0.001		p = 0.130		p = 0.598	

Supplementary Table 2 Legend:
Two-way ANOVA with Bonferroni post hoc test for multiple comparisons. Mean values are reported ± SEM with n listed in parentheses. P values below columns represent the comparison between the two above values; P values at the end of rows represent the comparison between the two values to the left.

bioRxiv preprint doi: https://doi.org/10.64898/2026.04.20.719713; this version posted April 23, 2026. The copyright holder for this preprint (which was not certified by peer review) is the author/funder, who has granted bioRxiv a license to display the preprint in perpetuity. It is made available under aCC-BY 4.0 International license.

Supplementary Table 3

2-Way ANOVA			
condition	control	IRAG 1-43-cit	IRAG vs. Control
HCN4 Δ1-25	-113.6 ± 2.4 (8)	-117.2 ± 1.8 (8)	p = 0.281
+ cAMP	-104.6 ± 2.2 (13)	-109.2 ± 1.8 (8)	p = 0.127
	p = 0.005	p = 0.022	

Supplementary Table 3 Legend:
Two-way ANOVA with Bonferroni post hoc test for multiple comparisons. Mean values are reported ± SEM with n listed in parentheses. P values below columns represent the comparison between the two above values; P values at the end of rows represent the comparison between the two values to the left.

bioRxiv preprint doi: https://doi.org/10.64898/2026.04.20.719713; this version posted April 23, 2026. The copyright holder for this preprint (which was not certified by peer review) is the author/funder, who has granted bioRxiv a license to display the preprint in perpetuity. It is made available under aCC-BY 4.0 International license.

Supplementary Table 4

2-Way ANOVA			
condition	control	IRAG 1-43-cit	IRAG vs. Control
HCN4 S719X	-116.6 ± 2.4 (11)	-112.5 ± 1.1 (15)	p = 0.062
+ cAMP	-106.5 ± 1.1 (9)	-107.4 ± 1.7 (9)	p = 0.709
	p < 0.001	p = 0.030	

Supplementary Table 4 Legend:
Two-way ANOVA with Bonferroni post hoc test for multiple comparisons. Mean values are reported ± SEM with n listed in parentheses. P values below columns represent the comparison between the two above values; P values at the end of rows represent the comparison between the two values to the left.

bioRxiv preprint doi: https://doi.org/10.64898/2026.04.20.719713; this version posted April 23, 2026. The copyright holder for this preprint (which was not certified by peer review) is the author/funder, who has granted bioRxiv a license to display the preprint in perpetuity. It is made available under aCC-BY 4.0 International license.

Supplementary Table 5

two-tailed t-test			
condition	control	IRAG 1-43-cit	IRAG vs. Control
HCN4 V604X	-101.9 ± 2.6 (10)	-107.1 ± 2.3 (9)	p = 0.160

Supplementary Table 5 Legend:
Two-tailed t-test between the two conditions. Mean values are reported ± SEM with n listed in parentheses. P value is reported in the final column.

bioRxiv preprint doi: https://doi.org/10.64898/2026.04.20.719713; this version posted April 23, 2026. The copyright holder for this preprint (which was not certified by peer review) is the author/funder, who has granted bioRxiv a license to display the preprint in perpetuity. It is made available under aCC-BY 4.0 International license.

Supplementary Table 6

2-Way ANOVA			
condition	control	IRAG 1-43-cit	IRAG vs. Control
HCN4 PT/AF	-119.8 ± 1.3 (9)	-120.5 ± 1.2 (10)	p = 0.671
+ cAMP	-108.3 ± 1.2 (9)	-104.3 ± 1.3 (9)	p = 0.026
	p < 0.001	p < 0.001	

Supplementary Table 6 Legend:
Two-way ANOVA with Bonferroni post hoc test for multiple comparisons. Mean values are reported ± SEM with n listed in parentheses. P values below columns represent the comparison between the two above values; P values at the end of rows represent the comparison between the two values to the left.

bioRxiv preprint doi: https://doi.org/10.64898/2026.04.20.719713; this version posted April 23, 2026. The copyright holder for this preprint (which was not certified by peer review) is the author/funder, who has granted bioRxiv a license to display the preprint in perpetuity. It is made available under aCC-BY 4.0 International license.

Supplementary Table 7

2-Way ANOVA			
condition	control	IRAG 1-43-cit	IRAG vs. Control
HCN2-4N VVGPT	-111.1 ± 2.1 (13)	-106.8 ± 1.7 (10)	p = 0.095
+ cAMP	-97.7 ± 1.35 (11)	-95.6 ± 1.3 (10)	p = 0.114
	p < 0.001	p < 0.001	

Supplementary Table 7 Legend:
Two-way ANOVA with Bonferroni post hoc test for multiple comparisons. Mean values are reported ± SEM with n listed in parentheses. P values below columns represent the comparison between the two above values; P values at the end of rows represent the comparison between the two values to the left.
